# The Cost of Waiting for a Transcatheter Aortic Valve Replacement in Medicare Beneficiaries With Severe Aortic Stenosis

**DOI:** 10.1016/j.shj.2024.100321

**Published:** 2024-06-12

**Authors:** Ankur Sethi, Sammy Elmariah, Candace Gunnarsson, Michael Ryan, Soumya Chikermane, Christin Thompson, Mark Russo

**Affiliations:** a Division of Cardiology, Department of Medicine, Robert Wood Johnson University Hospital, New Brunswick, New Jersey; b Division of Cardiology, Department of Medicine, University of California San Francisco (S.E.); c Gunnarsson Consulting, Jupiter, Florida; d MPR Consulting, Cincinnati, Ohio; e Edwards Lifesciences, Irvine, California; f Division of Cardiac Surgery, Rutgers-Robert Wood Johnson Medical School, New Brunswick, New Jersey

**Keywords:** Costs, Severe Aortic Stenosis, Transcatheter Aortic Valve Replacement

## Abstract

**Background:**

Aortic stenosis (AS) is a prevalent valvular disorder necessitating timely intervention, particularly when symptomatic. Aortic valve replacement (AVR) is the recommended treatment, but delays in access to AVR are common and linked to adverse outcomes and increased health care costs. This study aims to assess the health care cost burden associated with delaying transcatheter AVR (TAVR) in Medicare Advantage beneficiaries with clinically significant AS.

**Methods and Results:**

This retrospective database study utilized the Optum de-identified U.S. claims database, encompassing Medicare Advantage enrollees. Patients aged 65 years or older were identified as having AS based on medical billing codes and were required to have a record of syncope, dyspnea, fatigue, chest pain/angina, or heart failure prior to, on or within 30 days of their incident AS diagnosis. Total health care costs were analyzed over a 2-year period, regressed against the delay in receiving TAVR, and adjusted for covariates. In the 4105 patients meeting study inclusion criteria, delays in TAVR were associated with a significant increase in health care costs, translating to those waiting 12 months for TAVR incurring an additional cost of $10,080 compared to those receiving TAVR promptly. Non-TAVR related costs largely drove this increase.

**Conclusions:**

Delaying TAVR in clinically significant AS patients is associated with higher health care costs, emphasizing the need for timely interventions. Addressing delays in TAVR access and optimizing pre-TAVR workup can potentially improve patient outcomes and reduce health care expenditure.

## Introduction

Aortic stenosis (AS) is the most common valvular disorder requiring medical care and surgical or transcatheter interventions.^1^ When untreated, symptomatic, severe AS has a dismal prognosis with 35%-60% of patients dying within a year from the onset of symptoms.^2^, ^3^, ^4^, ^5^ Aortic valve replacement (AVR), either surgical AVR (SAVR) or transcatheter AVR (TAVR), is the only effective treatment recommended by American Heart Association/American College of Cardiology guidelines for symptomatic, severe AS patients.^6^ Nevertheless, even with broader indications since the advent of TAVR, it is estimated that up to two-thirds of patients with symptomatic, severe AS who are not offered treatment due to various reasons including incomplete heart team evaluation or misinterpretation of severity, are appropriate candidates for AVR.^7^^,^^8^ And even among those who are treated, for a variety of reasons, including failure to diagnose, delays in referral, patient hesitancy, and a lack of programmatic bandwidth to process patients, treatment is often delayed.

Previous studies have found that even a modest increase in the wait times for AVR may lead to a substantial increase in mortality ranging from 2% to 14%.^9^ A population-based analysis from Ontario, Canada, found TAVR wait times to be longer compared to SAVR and associated with higher mortality rate and hospitalization.^10^ A single-center study from the United States during the COVID-19 shutdown found that 10% of patients waiting for TAVR experienced a cardiac event during the first month, and 35% did so over the course of the next 3 months.^11^ In addition to increased mortality and adverse events, delay in care of severe AS patients may incur substantial costs. A cost-utility analysis from the perspective of the Spanish National Health Service, comparing immediate TAVR to wait time of 3 to 12 months, found eliminating wait time to be cost-effective with an incremental cost of €12,500/quality of life year gained; however, eliminating wait time led to substantial cost saving in patients with acute heart failure or syncope of approximately €6686/patient.^12^

The purpose of this analysis was to explore the incremental health care cost burden associated with delaying TAVR in Medicare Advantage (MA) beneficiaries with clinically significant AS. This study differs from previous ones because it uses comprehensive economic claims data to estimate the cost of delays in treatment.

## Methods

This is a real-world retrospective database study leveraging the Optum de-identified U.S. claims database, containing MA enrollees across the United States up to Quarter 3 of the year 2022. The data includes information on the health care services that are covered for beneficiaries enrolled in Medicare Parts A and B. Utilization for individual beneficiaries can be linked over time and across providers. Detailed information submitted by providers from claims-data includes, but is not limited to, the following: an encrypted beneficiary identifier and beneficiary responsibility; provider identity; Medicare program payments; from and through dates; admission and discharge dates; information on source of admission and discharge destination (including death) for institutional providers; International Classification of Diseases Ninth Revision (ICD-9) or 10th Revision (ICD-10) diagnosis and procedure codes; revenue centers, Healthcare Common Procedure Coding System/Current Procedural Terminology codes, and charges associated with those services; and annual demographic and enrollment information.

Optum applies a proprietary algorithm to determine standardized costs, which are inflated to the latest year of data. These costs include both medical and pharmacy claims. The medical claims contain data for inpatient and outpatient professional services including services such as outpatient surgery, laboratory, and radiology, and contain information specific to professional and facility claims. The pharmacy claims contain claims submitted by pharmacies for drugs dispensed on an outpatient basis.

All data used to perform this analysis were de-identified and accessed in compliance with the Health Insurance Portability and Accountability Act. As a retrospective analysis of a de-identified database, the research was exempt from institutional review board review under 45 CFR 46.101(b) (4).

### Study Population

The study population included patients aged 65 years or older with ​≥1 inpatient or ​≥2 outpatient diagnoses of AS using the ICD-10 diagnosis code “I35.0.” To improve specificity, a minimum of 6 months of continuous enrollment prior to their first diagnosis of AS was required to capture baseline comorbidities.^13^^,^^14^ Patients were also required to have a record of an echocardiogram either 6 months prior (baseline period) or on the same day/visit of their first diagnosis of AS to capture the incident diagnosis. To ensure that the patients had clinically significant AS, they were required to have a record of syncope, dyspnea, fatigue, chest pain/angina, or heart failure prior to, on or within 30 days of their incident AS diagnosis.

**Figure 1:**
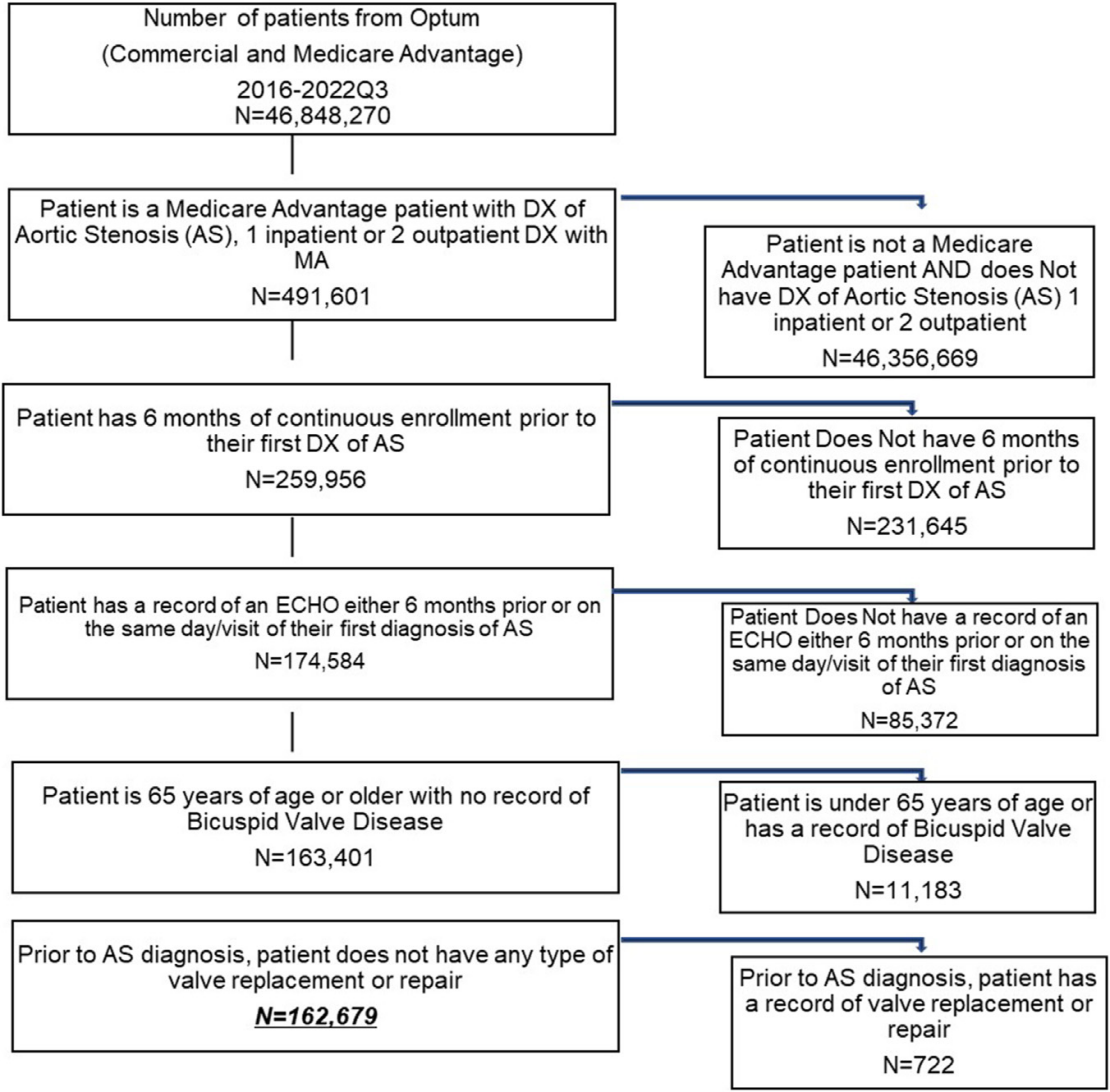
Consort diagram for Optum Medicare Advantage. Abbreviations: AS, aortic stenosis; DX, diagnosis; ECHO, echocardiography; MA, medicare advantage.

**Figure 2:**
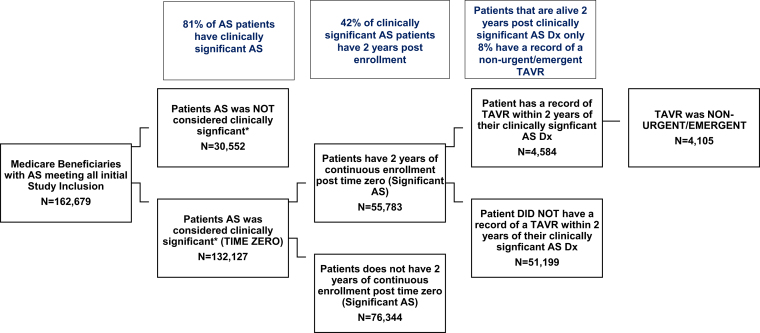
Defining study cohort Optum Medicare Advantage. Abbreviations: AS, aortic stenosis; DX, diagnosis; TAVR, transcatheter aortic valve replacement.

Patients considered to be having an urgent/emergent TAVR were excluded. These patients were defined as having their TAVR on the same day as time zero (clinically significant AS) or having a record of a nonelective TAVR within 1 to 90 days following time zero. Patients were excluded if they were <65 years of age on their first diagnosis of AS, since Medicare beneficiaries under the age of 65 are most often disabled. Additional exclusions were patients with a diagnosis of bicuspid valve disease (anytime) or a record of any type of valve replacement or repair prior to their AS diagnosis. Finally, patients had to have 2 years of continuous enrollment post-time zero (significant AS) and a record of a nonurgent TAVR anytime within the 2-year window from their clinically significant AS diagnosis. Figures 1 and 2 provide a consort diagram of the initial inclusion/exclusion criteria for this study and cohort attrition, respectively.

### Statistical Analysis

The outcome of the study was the additional cost burden (total cost) incurred (measured as dollars/unit time) for the delay in receiving an elective TAVR for beneficiaries with clinically significant AS. First, for each patient in the study, their total costs were aggregated over the 2-year follow-up period from their diagnosis of clinically significant AS. Since each study participant had to have a record of a nonurgent TAVR within the 2-year follow-up period, the cost of their TAVR procedure was included. Next, the total 2-year costs were regressed over the number of days from their clinically significant AS diagnosis to TAVR in a multiple linear regression model. Separate models were estimated with the following as model covariates: age, sex, race, region, coronary artery bypass graft, or percutaneous coronary intervention, coronary artery disease, other valve diseases, and the average Elixhauser Comorbidity Index (ECI), which identifies 31 categories of comorbidities associated with mortality. The *p* value less than 0.05 was considered statistically significant. The statistical analysis was conducted using the Statistical Analysis Software (SAS), v 9.4.

## Results

There were 491,601 Optum MA patients with at least 1 inpatient or 2 outpatient claims for AS from 2016-Q3 2022, respectively. After applying inclusion/exclusion criteria of a record of an echocardiogram, baseline enrollment, 65 years of age or older, no bicuspid disease, or a history of valve surgery, the total number of patients with AS included in the analytic cohort was 162,679 (see Figure 1).

The cohort attrition diagram (Figure 2) describes the additional criteria for the study cohort. Of these AS patients that met the initial inclusion, 81% (n = 132,127) had a record of clinically significant AS (sAS) defined as a record of syncope (15.70%), dyspnea (56.14%), fatigue (38.36%), chest pain/angina (5.69%), and/or heart failure (40.01%) prior to, on or within 30 days of their incident AS diagnosis. Patients were also required to have a minimum of 2 years of follow-up (continuous enrollment) from time zero (date of their sAS diagnosis), which reduced the size of the cohort by 58% (n = 55,783). Finally, patients were required to have a record of nonurgent/emergent TAVR procedures within the 2-year follow-up. Of the patients who were alive during 2 years post-sAS, only 7% (n = 4105) have a record of a nonurgent/emergent TAVR (see Figure 2).

Table 1 provides patient demographics and clinical characteristics for the study cohort. Average (SD) patient age was 76.17 years (5.71), and the majority were male (50.86%) and Caucasian (79.34%). The southern region had the most representation (34.45%). The average (SD) ECI score was 4.65 (2.84).

**Table 1:** Patient demographics and baseline clinical characteristics

Variables	Cohort N = 4105
Demographic characteristics	
Age, mean (SD)	76.17 (5.71)
Sex	
Female	2017 (49.14%)
Male	2088 (50.86%)
Race	
Caucasian	3257 (79.34%)
Black	305 (7.43%)
Asian	52 (1.27%)
Hispanic	343 (8.36%)
Unknown	148 (3.61%)
Region	
Northeast	657 (16.00%)
Midwest	965 (23.51%)
South	1414 (34.45%)
West	1069 (26.04%)
Clinical characteristics	
Elixhauser Comorbidity Index, mean (SD)	4.65 (2.84)
Elixhauser comorbidities	
Congestive heart failure	1154 (28.11%)
Cardiac arrhythmias	1565 (38.12%)
Valvular disease	1818 (44.29%)
Pulmonary circulation disorders	226 (5.51%)
Peripheral vascular disease	994 (24.21%)
Uncomplicated hypertension	3144 (76.59%)
Complicated hypertension	1000 (24.36%)
Paralysis	32 (0.78%)
Other neurological disorders	197 (4.80%)
Chronic pulmonary disease	1052 (25.63%)
Diabetes uncomplicated	594 (14.47%)
Diabetes complicated	1074 (26.16%)
Hypothyroidism	893 (21.75%)
Renal failure	1052 (25.63%)
Liver disease	134 (3.26%)
Peptic ulcer disease	50 (1.22%)
AIDS	∗
Lymphoma	56 (1.36%)
Metastatic cancer	37 (0.90%)
Solid tumor without metastasis	395 (9.62%)
Rheumatoid arthritis	253 (6.16%)
Coagulopathy	226 (5.51%)
Obesity	560 (13.64%)
Weight loss	148 (3.61%)
Fluid and electrolyte disorders	499 (12.16%)
Iron deficiency anemia	90 (2.19%)
Deficiency anemia	375 (9.14%)
Alcohol abuse	51 (1.24%)
Drug abuse	46 (1.12%)
Psychoses	∗
Depression	423 (10.30%)
Other comorbidities	
Coronary artery disease	1822 (44.38%)
Other valve disease	1249 (30.43%)
Previous PCI or CABG	67 (1.63%)

Abbreviations: AIDS, acquired immunodeficiency syndrome; CABG, coronary artery bypass graft; PCI, percutaneous coronary intervention.

∗ Counts less than 11 are suppressed. Data presented as mean (SD) or n (%), where applicable.

**Figure 3:**
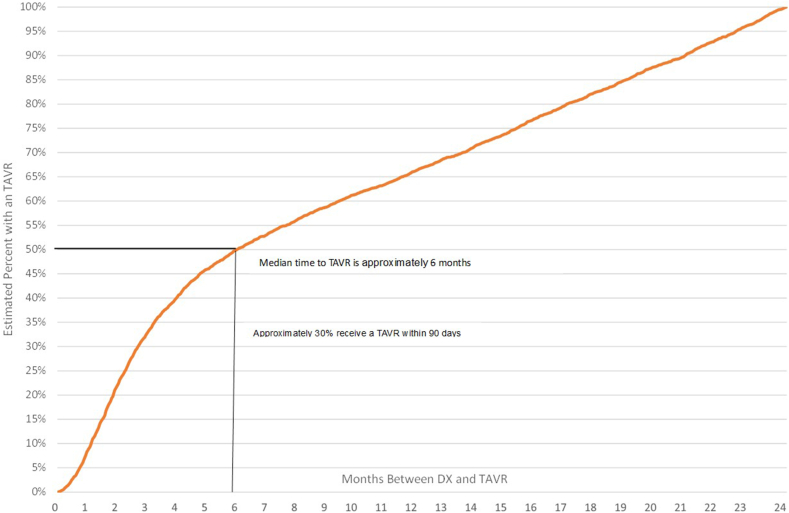
Time from a significant AS diagnosis to TAVR. Abbreviations: AS, aortic stenosis; DX, diagnosis; TAVR, transcatheter aortic valve replacement.

Patients in the study cohort had 2 years of continuous enrollment from the date of their sAS (time zero). Figure 3 provides a Kaplan-Meier curve of time from sAS to TAVR by month for the 2-year outcome period. Approximately 30% of patients received a TAVR within 90 days, 50% within 6 months, and approximately 64% within 12 months.

**Figure 4:**
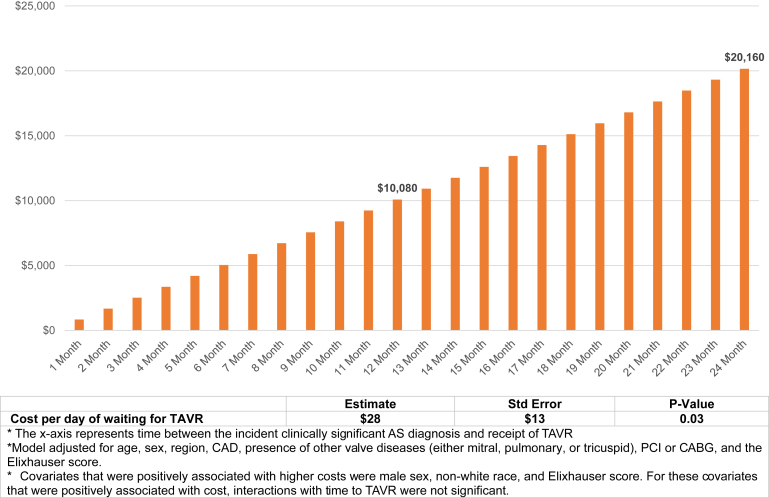
Multivariable regression results—cost of delay in care (difference in 2-year total cost). The x-axis represents time between the incident clinically significant aortic stenosis (AS) diagnosis and receipt of TAVR. Model adjusted for age, sex, region, coronary artery disease, presence of other valve diseases (mitral, pulmonary, or tricuspid), percutaneous coronary intervention or coronary artery bypass graft, and the Elixhauser score. Covariates that were positively associated with higher costs were male sex, non-White race, and Elixhauser score. For these covariates that were positively associated with cost, interactions with time to TAVR were not significant. Abbreviations: AS, aortic stenosis; TAVR, transcatheter aortic valve replacement.

When regressing time to TAVR on costs (over a 2-year outcome window), adjusting for patient demographics and comorbid conditions, patients with clinically significant AS have an associated $28/d increase in health care costs for every day a nonurgent/emergent TAVR is delayed. This equates to $840 a month, $10,080 a year, or $20,160 over 2 years (study time horizon) (see Figure 4). Other covariates that were positively associated with higher costs were male sex, non-White race, and Elixhauser score. For the covariates that were positively associated with cost (sex, race, and ECI score), interactions with time to TAVR were not significant.

When exploring these additional costs further in post-hoc analyses, they appear to be largely driven by non-TAVR costs. We subtracted the cost of the TAVR hospitalization from the total health care costs during 2 ​years to create two cost variables: (1) TAVR costs and (2) non-TAVR costs. Although there was no difference in incremental cost/day for TAVR index hospitalization (-$1.26, *p* = 0.85) or the cost of TAVR index hospitalization +30 days from discharge (-$0.76, *p* = 0.92), non-TAVR costs increased with increasing time to treatment ($29.19. *p* = 0.0035).

## Discussion

In this study of contemporary Optum MA beneficiaries with sAS that have a record of receiving a TAVR within the 2-year follow-up period, approximately 30% received TAVR within 90 days and up to 50% within 6 ​months of diagnosis of clinically significant AS. These delays in treatment were associated with higher costs during the 2-year follow-up period. The 2-year health care cost for a patient who received a TAVR at month 12 after diagnosis of sAS was $10,080 more than a patient who received a TAVR 1 ​month after their diagnosis, and $20,160 more for a patient who received a TAVR at month 24 compared to another who received it at month one after diagnosis. This underlines that waiting for a TAVR in sAS patients is associated with higher costs.

### Delay Is Associated With Worse Outcomes

AVR, with either TAVR or SAVR, is the only therapy known to improve outcomes in patients with symptomatic severe AS and has received a class I indication (level of evidence A) by the American Heart Association/American College of Cardiology guidelines.^6^ However, despite the strong recommendation, previous studies have reported mortality rate ranging from 2% to 14% during the wait period for AVR possibly related to the characteristics of the patients studied: age, surgical risk, and prevalence of comorbidities like functional class, heart failure, and left ventricle function.^9^ Despite the expansion of TAVR indications, the gap between those who are eligible for an AVR and those who receive an AVR does not seem to be closing significantly. Delaying TAVR for 6 months impacted the 2-year overall survival in patients that were either intermediate risk (0.81 for prompt TAVR vs. 0.67 for delayed TAVR) or low risk (0.95 for prompt TAVR vs. 0.85 for delayed TAVR).^15^

### Factors That Could Contribute to Delays or Reduce Access to TAVR

One of the striking findings of our analysis is that almost 50% of patients during 2016-Q3 2022, in the United States, waited 6 ​months or more for TAVR after the diagnosis of clinically significant AS (Figure 3). A population-based study from Ontario, Canada, during 2010-2016 reported a median wait time of 80 days from the referral to TAVR.^16^

It is possible that the wait time for TAVR in our study was impacted by the effect of the COVID-19 pandemic on the health care delivery in the United States. However, COVID was only relevant for two-fifths of the study period. Also, a single-center study from the United Kingdom reported no difference in median wait time from referral to TAVR between the pre- and peri-COVID-19 pandemic periods (100 vs. 124 days; *p* = 0.9).^17^

Although our study did not address mechanisms or causes of delay in care, existing research suggests these delays may result from referral patterns, recognition of disease, patient preferences, as well as structural issues in the health care system. Limited access to TAVR, specifically, is mediated by multiple factors including hospital size, location, and ownership status. A study using data from the National Readmission Database 2012-16 found that not-for-profit, large, urban, teaching hospitals had higher use of TAVR than investor-owned, government-owned, small, or nonurban hospitals.^18^ Although, with expansion of TAVR, the majority of patients now live within a health care referral region, the travel time is significantly longer for patients living in rural areas and the Midwest or Southern United States.^19^^,^^20^ sAS treatment disparities in underserved minorities based on patient related factors and health care system related factors are well recognized.^21^ It appears that TAVR may have helped to bridge the gender disparity in the treatment of sAS; however, more work is needed to address the racial disparity.^22^^,^^23^

### Next Steps for Reducing Delay in Care

Among clinically significant AS patients, waiting for TAVR increases health care costs. A safe wait time from the incident diagnosis to TAVR remains unknown. It likely varies by severity and type of clinical symptoms, extent of cardiac damage from AS, and other comorbidities.^24^ Further research should focus on identifying the high-risk patients who are at risk for short-term mortality and morbidity in the absence of timely TAVR. Considering AS is typically an indolent valvular disorder, some of the delay is likely contributed by the delay in diagnosis of AS at an earlier stage. The current study focuses on patients with a billing code for AS, indicating that AS has been diagnosed and recorded in these patients; however, another study has shown that a significant proportion of patients (15%) with severe AS (identified using echocardiography) did not receive a diagnosis of AS up to a year from the echocardiography.^25^ Patients with sAS usually undergo workup including echocardiogram, additional imaging, computerized tomographic scan, and heart team evaluation in preparation for TAVR. Each of these steps has the potential to cause a delay in TAVR for patients who otherwise have limited access to care.^20^ Some of the lessons learned during the COVID pandemic can be valuable in streamlining the pre-TAVR workup and shortening the delay.

### Limitations

In this current study, the analysis was restricted to patients who received a TAVR and survived a 2-year follow-up period to ascertain the impact of delay in TAVR on health care utilization costs from the health care payer’s perspective. Since it may sometimes be difficult to ascertain from population-based studies if patients who died during waiting period would have been considered appropriate for TAVR by heart team evaluation, we purposefully excluded patients who did not receive TAVR for 2 years to avoid overestimating the health care utilization by sAS patients who otherwise would not be candidates for TAVR. We used ICD codes to ascertain the diagnosis of clinically significant AS, inclusion and exclusion criteria, and comorbidities using an administrative database, which could potentially introduce bias due to under- or over-coding. Because of the nature of the database, we could not control for imaging or laboratory variables, or provider preference on the outcomes.

## Conclusions

Among contemporary MA with clinically significant AS, approximately 50% of patients wait for their TAVR for at least 6 months from when they were diagnosed with clinically significant AS, and delay in TAVR is associated with a significantly high cost during the 2-year follow-up period.

## Ethics Statement

As this was a noninterventional, retrospective, observational study that collected de-identified data for patients who met eligibility criteria, informed consent was not required from patients under an institutional review board exemption status. All aspects of this study were conducted in compliance with the Health Insurance Portability and Accountability Act of 1996 regulations and the act’s Omnibus Rule of 2013.

## Funding

This study was sponsored by 10.13039/100006520Edwards Lifesciences.

## Disclosure Statement

S. Elmariah has received research grants from Edwards Lifesciences, Medtronic, and Abbott, received consulting fees from Edwards Lifesciences, and has served as a consultant or advisor for Edwards Lifesciences. C. Gunnarsson and M. Ryan are paid consultants to Edwards Lifesciences. M. Russo reports administrative support, statistical analysis, and writing assistance were provided by Edwards Lifesciences Corporation; has received research grants from Edwards Lifesciences; and has served as a consultant or advisor for Abbott, Boston Scientific, and Edwards Lifesciences. S. Chikermane and C. Thompson are Edwards Lifesciences employees. The other author had no conflicts to declare.
